# The role of resilience and coping behavior in schizophrenia in the prevention of psychosis relapse

**DOI:** 10.1192/j.eurpsy.2024.1553

**Published:** 2024-08-27

**Authors:** V. Mitikhin, M. Kuzminova, L. Alieva

**Affiliations:** Department of Mental Health Services, Mental Health Research Centre, Moscow, Russian Federation

## Abstract

**Introduction:**

According to scientific sources, personal resources in the form of resilience, stress coping skills play an important protective role in the prevention of psychosis recurrence. Weakening the psychological capacity of the patient and increasing stress are risk factors for psychosis.

**Objectives:**

To study the protective role of such personal resources as resilience and coping with stress in schizophrenia, as well as the influence of negative symptomatology and psychosociorehabilitation intervention on these factors.

**Methods:**

Clinical-psychopathological, statistical, and psychometric methods were used (Alfimova-Golimbet’s resilience scale, Amirkhan’s coping strategies questionnaire, and PANSS). Patients of two groups participated in the study: 1 - members of a community organization (OO), n=49, who in addition to psychopharmacotherapy were given comprehensive long-term psychosociorehabilitation (3.7±2.5 years), 2 - patients of the medical-rehabilitation department of a psychiatric hospital (MRO), n=48, in whom the psychosociorehabilitation intervention was shorter (40.3±6.5 days).

**Results:**

The results of the study showed that significant predictors of a favorable course of the schizophrenic process were high indicators of resilience, coping behavior, and a small degree of negative symptomatology. Analysis of the data regarding patients’ coping with stress shows that constructive coping strategies are more frequent in both groups. Thus, “problem solving” (24.3 points in GS and 22.9 points in MPO) and “search for social support” (23.0 points and 22.7 points, respectively), that is patients of both groups are generally oriented to a productive way of coping with difficult situations and are ready to seek help from others in a difficult situation. Notably, the strategy of “problem avoidance” is less pronounced (18.5 points and 19.4 points, respectively). The high resilience scores in the GS group (32.5 points), comparable to the norm in the population (33.1 points), are explained by long-term comprehensive psychosociorehabilitation, while the resilience scores in the MPO group are lower - 28.7 points. Negative symptoms of schizophrenia were equally pronounced in both groups, manifested by difficulties in communication (2.6 points each), passive-apathetic social withdrawal (2.7 points each). Such negative symptoms as blunting of affect and emotional indifference were more pronounced in the MPO group - 3.2 points each vs. 2.8 points in the group from the GS.

**Conclusions:**

High levels of resilience and ability to cope with stress as a result of psychosociorehabilitation intervention allow patients to overcome difficult life circumstances more flexibly. They are associated with less pronounced negative symptoms, which generally helps prevent psychosis relapses and contribute to a more favorable course and prognosis of schizophrenia.

**Disclosure of Interest:**

None Declared

